# A Brief Account of a Fever Which Prevailed in the Neighborhood of Charlotte, Tennessee, during the Summer of 1846

**Published:** 1848-03

**Authors:** James M. Larkins

**Affiliations:** Charlotte, Tennessee


ART. II.—*A brief Account of a Fever which prevailed in the neighborhood of Charlotte, Tennessee, during the Summer of* 1846. By JAMES M. LARKINS, M.D., of Charlotte, Tennessee.

Charlotte stands on the conglomerate, or red sandstone, which contains the iron ore worked on the Cumberland river. The surface of the country is rolling, and from the hill sides issue innumerable springs of fine limestone water, this rock underlying the sandstone which forms the matrix of the iron ore. To the eye, no source of malaria appears. No ponds of stagnant water exist, the streams have good currents, and there is no marshy ground in this district of country.

The summer of 1846 was hot and rainy, the thermometer rising repeatedly as high as 90°, and for many days in July and August ranging from 80° to 85°. Thunder storms were unusually frequent, and often violent. We counted five or six trees which had been struck by lightning during a single one of these storms in the neighborhood of Charlotte.

Of the fever which made its appearance in the midst of this weather, the following were the most prominent symptoms:—Pulse but little altered from its natural state, soft, compressible, and only slightly increased in volume; cheeks flushed; countenance not expressive of pain or disease; intolerance of light; skin soft. The patient generally lay on his back, with his legs drawn up in the bed, and his eyes closed; but the latter was not a uniform attendant, some of the patients sleeping but little. In nearly all, the sense of hearing was impaired. There was neither craving nor loathing of food, but when presented the patient was generally disposed to partake of it. The tongue, at the access of the disease, was pointed, and covered with a white fur; but in the progress, it becomes rough, chapped, and coated with black, and sordes also appeared on the teeth. The patient, after the fever was fully established, never complained of pain, and invariably, when asked how he was, answered that he was better. Generally on the second or third day some abatement of the symptoms was experienced, but after this the disease progressed, without any appreciable intermission, until the eighteenth, and in some instances, the twenty-fifth day.

The parotid glands suffered enlargement in many cases, and frequently suppurated. It was during the stage of the disease when this suppuration was going on that the patients were in greatest danger. If neglected, or improperly treated, not a few died at this period. The duration of the fever, from its access to the stage of decided convalescence, was generally about six weeks.

*Treatment.*—The circulation being but slightly affectod, bleeding was not often practiced, and when resorted to the effects were not satisfactory. Emetics were rarely given, and, except where the case was of a clearly bilious character, did not appear to be beneficial. My favorite remedy was a combination of calomel and ipecac., generally three grains of each, repeated according to circumstances. When given as often as once in three hours, this dose seldom excited nausea or vomiting, and ptyalism very rarely followed; but a relaxation of the surface took place, and subsequently, gentle action of the bowels. As a refrigerant, nitrate of potash was administered, and when the disease manifested a local tendency blisters were resorted to with advantage. Cold water was freely applied to the head during the inflammatory stage of the fever, and the relief afforded by it was manifest.

The acute sufferings of the patient commenced with the secondary symptoms; namely: with the inflammation of the parotids. For these, emollient poultices were applied, and the matter was evacuated as soon as the tumors began to point. During the suppurating stage it was necessary to support the patients by tonics, stimulants, and nourishing diet. The vegetable bitters were administered in conjunction with wine and brandy. Our indigenous tonics, prunus Virginiana, cornus Florida, and liriodendron tulipifera, were found equal to any others.

In looking back over the history of this endemic, I am not able to recall to mind a single case which seemed to have been arrested by medical treatment, and I am not sure that the effect of treatment was in any instance to bring the malady to a speedier termination than it would have attained without it. But while I admit thus much, I am fully persuaded that the effect of treatment was to conduct to a happy termination many cases which, left to themselves, would have had a fatal issue. This opinion rests upon the fact that many patients who took no medicine died, and that a fatal termination was rarely witnessed where judicious medical aid was enjoyed by the sick.

This fever was evidently of the typhoid type, and it is worthy of remark that this form of fever seems to be taking the place, to a great extent, of the bilious remittent fever which prevailed so long in all parts of the Mississippi valley. Typhoid fever would appear to be pursuing a steady, though slow march, from the northern to the southern parts of the United States. Long after it had been the endemic of the New England States it was unknown in Tennessee. This change involves very grave consequences, for if the practitioner attempts to treat the new form of fever according to the system adapted to our former endemics, he will be unsuccessful. The lancet and quinine he finds inadmissible, and in truth the physician can do but little more than palliate symptoms, and support the patient through the disease.

Charlotte, March, 1847.

